# IgG4-related chronic sclerosing sialadenitis in a child with recurrent parotitis: a case report

**DOI:** 10.1186/s12887-021-03004-4

**Published:** 2021-12-20

**Authors:** Fabio Timeus, Mario Michele Calvo, Anna Maria Caci, Giorgio Oliviero Gallone, Federico Vittone

**Affiliations:** 1 Pediatric Department, Chivasso Hospital ASLTO4, Corso Galileo Ferraris 3, 10034 Chivasso, Italy; 2 ENT Department, Chivasso Hospital ASLTO4, Corso Galileo Ferraris 3, 10034, Chivasso, Italy; 3 Department of Pathology, Ivrea Hospital ASLTO4, Piazza Credenza 2, 10015, Ivrea, Italy

**Keywords:** IgG4-related disease, Chronic sclerosing sialadenitis, Juvenile recurrent parotitis, Case report

## Abstract

**Background:**

IgG4-related disease (IgG4-RD) includes a group of immune-mediated diseases histologically characterized by lymphoplasmacytic infiltrate with a prevalence of IgG4-positive plasma cells, storiform fibrosis and obliterative phlebitis. Autoimmune pancreatitis, sialadenitis, dacryoadenitis and retroperitoneal fibrosis are the most frequent manifestations. IgG4-related sialadenitis usually affects submandibular glands and is very rare in children.

Here we report the case of IgG4-related sialadenitis in a six-year-old patient previously diagnosed as juvenile recurrent parotitis.

**Case presentation:**

A six-year-old patient was referred to our Centre for left parotid swelling of 4 × 3 cm, that was tender, soft in consistency, with overlying red and warm skin. His general condition was good but he was subfebrile; general examination revealed mild enlargement of left cervical lymph nodes. In the last 2 years he had had five episodes of parotitis, diagnosed by another pediatric Center as juvenile recurrent parotitis.

On ultrasound examination the left parotid gland appeared enlarged, inhomogeneous, with a colliquative intraparotid lymph node and no evidence of sialolithiasis. Laboratory tests showed an increase of white blood cells and anti-VCA IgM and IgG positivity, with anti-EBNA e anti-EA I negativity.

The patient was initially treated with oral antibiotics, but after 10 days the parotid became fluctuating, requiring surgical biopsy and drainage. Postoperative course was regular, with complete remission under oral antibiotic and steroid therapy. Microbiological tests, including cultures for aerobic and anaerobic bacteria, mycobacteria and Bartonella, were negative.

Surprisingly, histology showed marked fibrosis and histiocytic and lymphoplasmacellular infiltrate with polyclonal plasma cells mostly expressing IgG4 immunoglobulins. Thus, the diagnosis of IgG4 related chronic sialadenitis in recurrent parotitis and recent EBV infection was made.

**Conclusions:**

IgG4-related sialadenitis is very unusual in children. Histology plays a key role in diagnosis, considering that up to 30% of patients have normal serum IgG4 levels, as shown in our case.

The lack of previous histological data makes it impossible to attribute our patient’s previous episodes of parotitis to IgG4-RD, though it is a very consistent possibility.

## Background

IgG4-related disease (IgG4-RD) emerged over the last two decades as an immune-mediated condition that unifies multiple fibro-inflammatory single-organ diseases previously considered as separate entities [1, 2]. A significant proportion of cases previously diagnosed as Miculicz’s disease (dacryoadenitis and enlargement of parotid and submandibular glands) and Kuttner tumor (chronic sclerosing sialadenitis of submandibular gland) are now considered IgG4-RD.

Diagnosis is based on clinical, serologic and typical histological findings. IgG4-RD histology shows a lymphoplasmacytic infiltrate with a prevalence of IgG4-positive plasma cells, storiform fibrosis and obliterative phlebitis [3, 4]. The majority of patients have high serum levels of IgG4, though up to 30% of reported patients do not [5, 6].

The pathogenesis is immune-mediated, with a central role of T-lymphocytes, whereas IgG4 do not seem responsible for the tissue damage [7, 8].

Autoimmune pancreatitis, sialadenitis, dacryoadenitis and retroperitoneal fibrosis are the most frequent manifestations of IgG4-RD. IgG4-related sialadenitis usually involves submandibular glands, whereas parotid glands are rarely affected [9, 10].

The natural history, without treatment, is the evolution towards advanced fibrosis, atrophy and loss of function. In the case of IgG4-related sialadenitis, xerostomia is a common evolution of the disease.

Immunosuppressive treatment is the mainstem of therapy. Steroids, usually prednisone, are commonly used as first-line agents, with good response rates. Methotrexate, azathioprine and mycophenolate have been used in relapsed-resistant cases [11–13]. Rituximab has a role in multi-resistant cases [14]. IgG4-RD requires a careful follow up for early detection of complications and/or involvement of other organs.

IgG4-related sialadenitis usually affects submandibular glands and is very rare in children [15–17].

Here we report the unexpected case of IgG4-related sialadenitis in a 6 years old patient previously diagnosed as juvenile recurrent parotitis.

## Case presentation

A six-year-old Caucasian patient was referred to our Centre for left parotid swelling.

His family history was negative for autoimmune diseases. In the last 2 years, his past history showed five episodes of parotitis that have been investigated by a regional Children’s Hospital. Once immunological and rheumatological pathologies were excluded, a diagnosis of juvenile recurrent parotitis was made. Careful oral hygiene and periodic otorhinolaryngological follow up were recommended.

General examination showed good general condition, T 37.5 °C, a parotid swelling of about 4 × 3 cm that was tender, soft in consistency with red and warm overlying skin, and a mild enlargement of left cervical lymph nodes (maximum 2 cm).

White blood cells (WBC) were 14,400/fL with 57% neutrophils, hemoglobin and platelet counts were normal. C-reactive protein, procalcitonin, electrolytes, glucose, creatinine, transaminases were normal, as well as amylase and lipase. Serology for Ebstein Barr virus (EBV) tested positive for IgM and IgG anti-VCA (S-anti VCA IgM 87 U/mL; S-anti VCA IgG 24 U/mL), with negative anti-EBNA IgG e anti-EA IgG. Serologies for Cytomegalovirus (CMV), Bartonella, Toxoplasma, Mantoux TST and Quantiferon-TB were negative. Serum IgG, IgA, IgM levels were normal (respectively 12.8, 1.4, 1.7 g/L) as well as serum IgG1, IgG2, IgG3, IgG4 (respectively 993, 141, 85, 49.3 mg/dL), whereas IgE level was high (752 kU/L). The left parotid gland appeared enlarged and inhomogeneous to ultrasound examination, with a colliquative intraparotid lymph node and an associated mild left latero-cervical lymphadenopathy. There was no evidence of sialolithiasis.

**Fig. 1:**
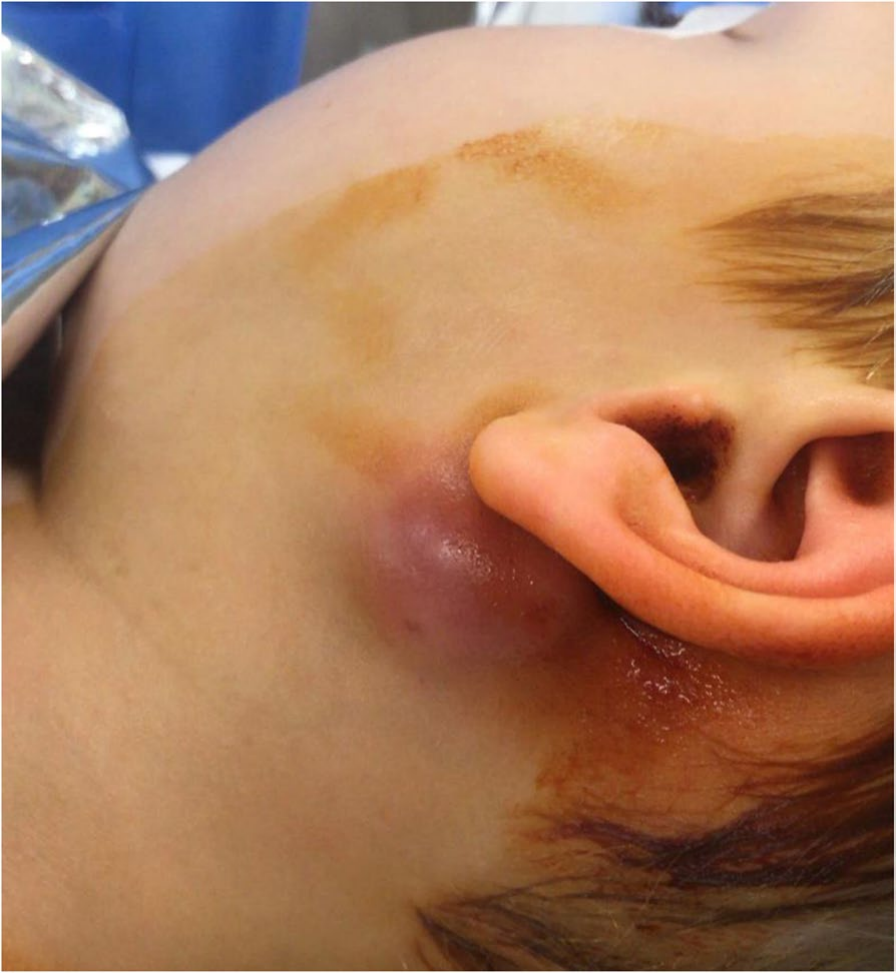
Left parotid swelling before surgery

The patient was first treated with ceftriaxone, then, after consultation with the infectious disease specialist, with cefixime and azithromycin. Despite antibiotic therapy, though, in 10 days the parotid gland became fluctuant, requiring surgical drainage and biopsy (Fig. 1). The patient was discharged 3 days after surgery without any complications, and treated with a further course of cefpodoxime (better tolerated than cefixime by the patient) and betamethasone with good clinical response.

Cultures for aerobic and anaerobic bacteria were negative as well as PCR and cultures for mycobacteria and PCR for Bartonella.

**Fig. 2:**
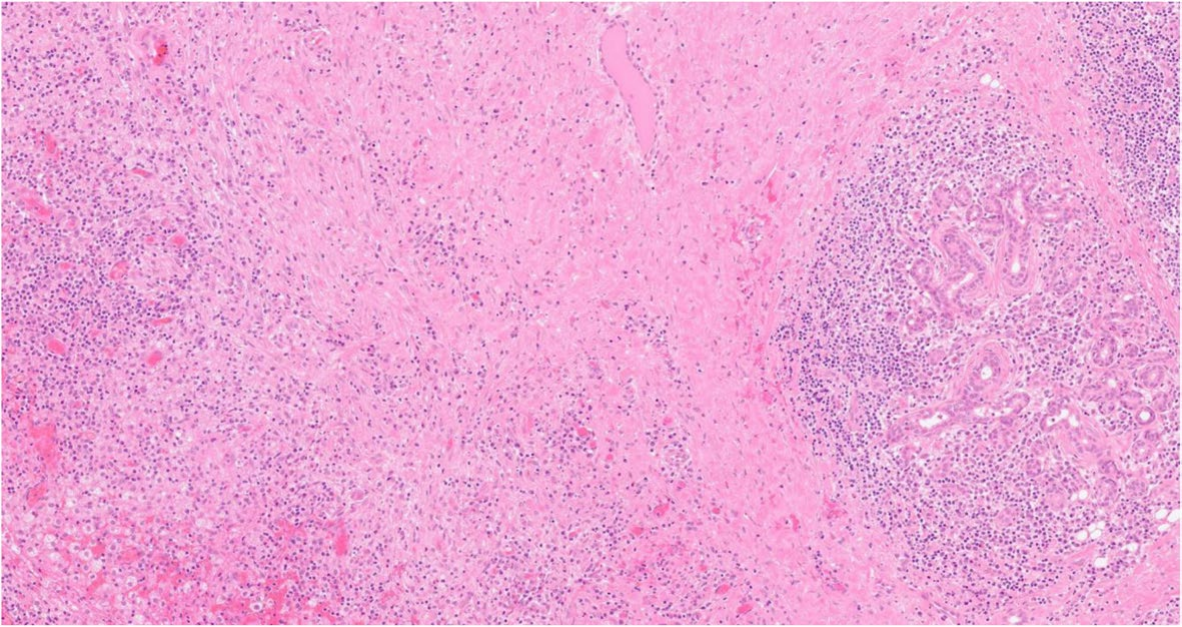
Interlobular fibrosis with dense lymphoplasmacellular infiltrate (hematoxylin and eosin, 10x magnification)

**Fig. 3:**
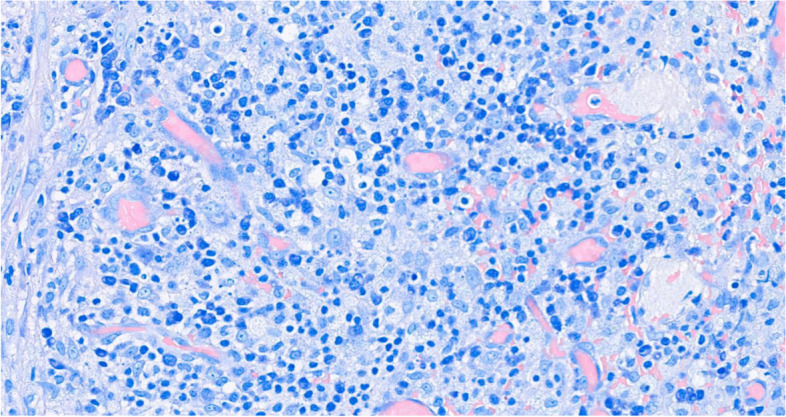
High number of plasma cells (Giemsa, 40x magnification)

**Fig. 4:**
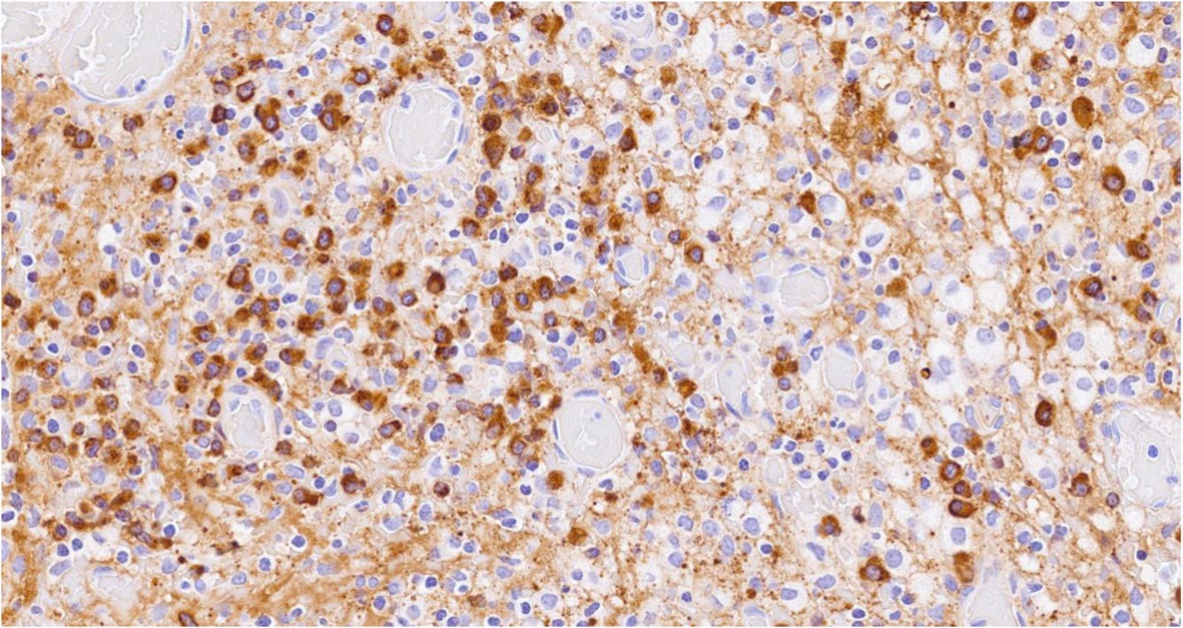
Plasma cell with strong cytoplasmic expression for IgG (IgG immunohistochemistry, 40x magnification)

**Fig. 5:**
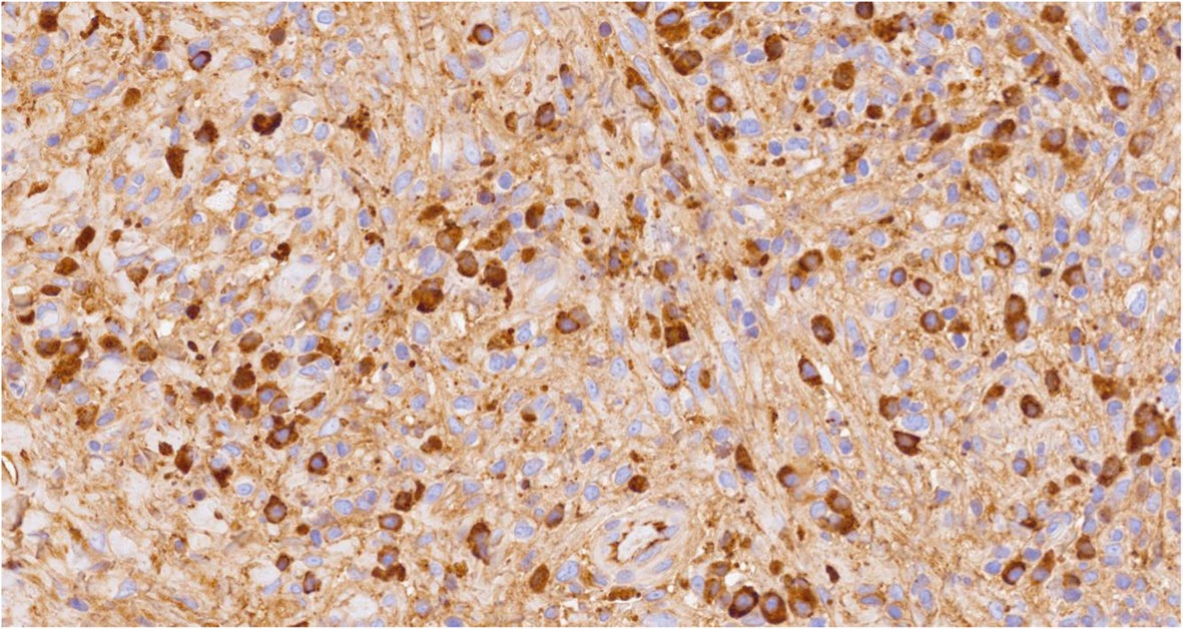
Plasma cell with strong cytoplasmic expression for IgG4 (IgG4 immunohistochemistry, 40x magnification)

**Fig. 6:**
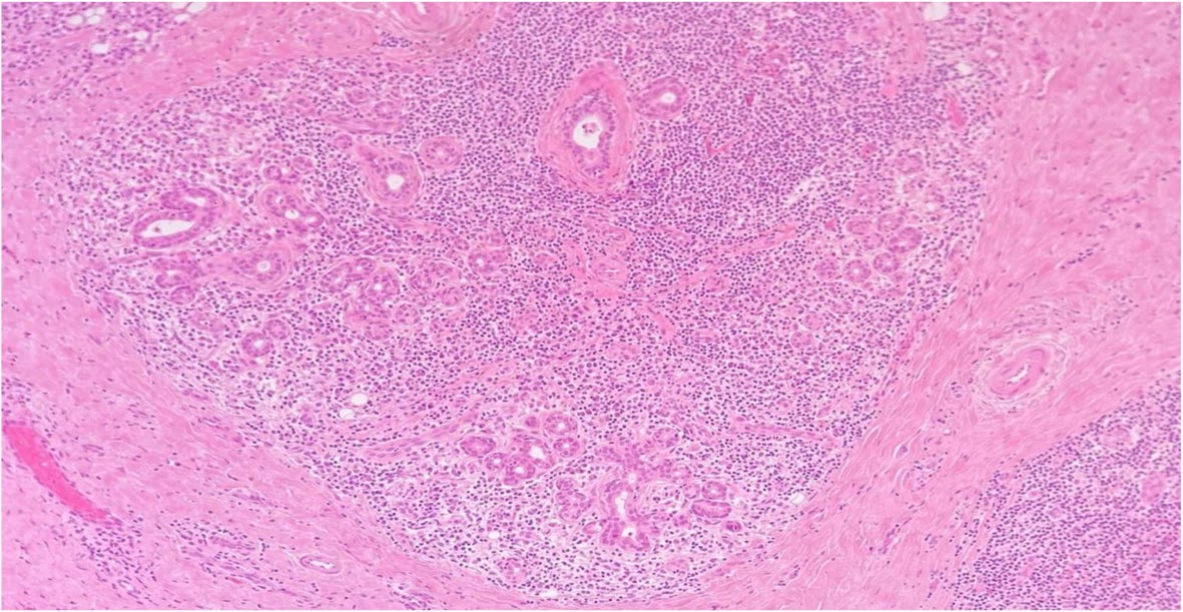
Interlobular fibrosis with dense infiltrate that disrupts and cancels the residual glandular structures (EE 10x)

Histology showed a salivary gland fragment containing marked interlobular fibrosis and dense intralobular lymphoplasmacellular infiltrates; some follicles showed a reactive germinal center. Peripheral fibrosis showed marked histiocytic and lymphoplasmacellular infiltration. The plasma cells were polyclonal (as defined by the balanced expression of kappa and lambda light chains), mostly expressing class G immunoglobulins. The IgG4 subclass meanly represented 50–60% of the total IgG, reaching up to 100 plasma cells/high-power field (HPF) in specific areas. (Figs.1, 2, 3, 4, 5 and 6).

The EBER-ISH reaction (Epstein Barr virus-encoded RNA in CISH, chromogenic in situ hybridization) was negative.

Thus, the final diagnosis was IgG4-related chronic sclerosing sialadenitis in recurrent parotitis and recent EBV infection.

The patient is now under strict follow up, and screening for other IgG4-related clinical manifestations is currently negative. Serum IgG, IgM, IgA are normal, as lymphocyte subpopulations. Antinuclear antibodies (ANA), anti Ro/SSA, anti-La/SSB, anti-double stranded DNA (anti-dsDNA Ab) are negative. FT3, FT4, TSH are normal. Anti-EBNA IgG antibodies are now positive (239 U/mL), as well as anti-VCA IgG (42 U/mL). Anti-VCA IgM are negative.

One year after hospitalization, the patient only had two other mild episodes of tender swelling of the left parotid gland with normal blood tests, successfully treated with a short course of amoxicillin and betamethasone, without adverse events. Considering the prompt clinical response, no long-term steroid therapy was proposed so far.

The parotid gland, though, persists enlarged (AP diameter 15 mm) with marked inhomogeneity at US examination.

## Discussion

The consensus statement [18] for histological diagnosis of IgG4-RD allows the distinction between lesions that are “histologically highly suggestive” and those that show “probable histological features”. In both cases, the presence of at least one between dense lymphoplasmacytic infiltrate, storiform fibrosis and obliterative phlebitis must be present. The IgG4/IgG ratio has to be at least 40%, and the number of plasma cells for HPF has to be specified.

According to Despande et al., our patient was classified as “histologically highly suggestive” (IgG4 / IgG > 40%, dense lymphoplasmacytic infiltrate, marked interlobular fibrosis and > 100 IgG4 / HPF). We did not observe storiform fibrosis nor obliterative phlebitis, rarely described in IgG4-related parotitis [18].

As mentioned before, our patient had had four previous episodes of parotitis, formerly diagnosed as “juvenile recurrent parotitis”. The lack of previous histological data makes it impossible to disprove this diagnosis and attribute those episodes to IgG4-RD instead, though it is a very consistent possibility.

Juvenile recurrent parotitis, in fact, is a recurrent non-obstructive and non-suppurative parotid inflammation. The highest incidence is between 3 and 6 years of age, spontaneously disappearing in the second decade of life [19–21]. Various viral agents, poor oral hygiene, congenital malformations of the salivary duct, immunological defects have been related to the recurrent episodes, but the etiology remains unknown in most cases. The possible association with a selective IgA deficiency has been described [22, 23]. Operative sialendoscopy may play a role in treatment, but the high rate of spontaneous remission allows a “wait- and-see” approach [24].

A very confounding feature of our case was the parotid gland appearance. The tender consistency with red and warm skin seemed more consistent with an infectious etiology at first examination, especially when compared to typical aspects of IgG4-related sialadenitis, which is elastic or firm, painless and persistent. In the hypothesis of a reactive infectious form, antibiotics were initially prescribed. Negative bacteriological and cultural examinations do not rule out the possible bacterial superinfection, as they were performed on the bioptical specimen, after 10 days of antibiotic therapy.

During the follow-up, the persistent enlargement of the left parotid with marked ecostructural inhomogeneity suggests a chronic process, with episodic reactivations, controlled on demand by corticosteroid therapy.

For what concerns laboratory tests, serum IgG4 were normal in our case, as described in a minority (30%) of cases [6, 25–27], whereas IgE were high, as frequently described in IgG4-RD.

Strehl JD et al [28] published a paper about non-specific chronic inflammatory conditions characterized by the presence of tissue infiltration of IgG4-positive plasma cells. A non- specific increase of IgG4-positive plasma cells was observed in rheumatoid synovitis, oral cavity lesions, and carcinoma-associated inflammatory responses. In sialadenitis caused by sialolithiasis a mean of 3 IgG4-positive plasma cells/hpf was observed versus a mean of 40/hpf in IgG4-related sclerosing sialadenitis. These data are consistent with our case.

Literature data show a relationship between EBV infection and increased IgG4 production. Takeuchi et al. observed an increase in EBV-infected cells in patients with IgG4-related lymphadenopathy by in situ hybridization [29]. Nagata et al. demonstrated in a subset of patients with Graves’ disease that EBV reactivation promotes IgG4 production. By in situ hybridization they observed the coexistence of EBER1-positive cells and IgG4 positive plasma cells in the same areas of thyroid tissue. However, no fibrosis nor obliterative phlebitis was present, thus configuring a condition that is distinct from an IgG4-RD. [30]

In our case, the laboratory demonstrated an acute/recent EBV infection. However, the negativity of the in situ hybridization for EBV leads us to believe that a causal link between EBV infection and the histological picture is unlikely.

## Conclusions

The lack of previous histological data makes it impossible to attribute our patient’s past episodes of parotitis to IgG4-RD, though it is a very consistent possibility. The present case, instead, fulfills the current histologic diagnostic criteria for IgG4-RD.

The histological finding was quite unexpected, as IgG4-related disease is rare in children, but it underlines once more the key role of the pathologist in the differential diagnosis of lymphadenopathies and masses of head and neck area.

Our findings are relevant for pediatricians to make a correct diagnosis and to set up an appropriate follow up.

### Reference values of our lab

EBV virus:

S-anti VCA IgM U/mL < 20 negative, 20–40 doubtful, > 40 positive.

S-anti VCA IgG U/mL < 20 negative, ≥20 positive.

S-anti EBNA IgG U/mL < 5 negative, 5–20 doubtful, ≥20 positive.

S-anti EA IgG U/mL < 10 negative, 10–40 doubtful, > 40 positive.

Immunoglobulins, 6 years:

S-IgG 5.6–1.5 g/L.

S-IgA 0.5–2.2 g/L.

S-IgM 0.3–1.2 g/L.

S-IgG1 400–1000 mg/dL.

S-IgG2 80–250 mg/dL.

S IgG3 20–96 mg/dL.

S-IgG4 3–108 mg/dL.

S-IgE < 100 kUI/L.

## Data Availability

The datasets used and/or analysed during the current study are available from the corresponding author on reasonable request, anonymously.

## Abbreviations

ANA: Antinuclear antibodies
anti-dsDNA Ab: Anti-double stranded DNA
CMV: Cytomegalovirus
EA: Early antigen
EBER - ISH reaction: Epstein Barr virus-encoded RNA in CISH, chromogenic in situ hybridization
EBNA: Epstein-Barr nuclear antigen
EBV: Ebstein Barr virus
EE: Hematoxylin-eosin
HPF: High-power field
IgG4-RD: IgG4-related disease
SS: Sjӧgren’s syndrome
TB: Tuberculosis
TST: Tuberculin skin testing
VCA: Viral capsid antigen
WBC: White blood cells
